# Transplanting Against the Gradient: Invasive Evaluation of Resting and Dynamic Left Ventricular Outflow Tract (LVOT) Obstruction in a Liver Transplant Candidate

**DOI:** 10.7759/cureus.94656

**Published:** 2025-10-15

**Authors:** Hugh Slifirski, Laurence Weinberg, Nattaya Raykateeraroj, Jemin A Suh, Anoop N Koshy, Dong-Kyu Lee

**Affiliations:** 1 Department of Anesthesiology, Austin Health, Melbourne, AUS; 2 Department of Critical Care, The University of Melbourne, Melbourne, AUS; 3 Department of Anesthesiology, Faculty of Medicine, Siriraj Hospital, Mahidol University, Bangkok, THA; 4 Department of Cardiology, Austin Health, Melbourne, AUS; 5 Department of Anesthesiology and Pain Medicine, Dongguk University Ilsan Hospital, Goyang, PRK

**Keywords:** diagnostic work up, fluid responsiveness, left ventricular outflow tract (lvot) obstruction, liver transplantation, orthotopic liver transplantation, risk stratify

## Abstract

Dynamic left ventricular outflow tract (LVOT) obstruction remains a clinically significant perioperative risk factor in candidates for liver transplantation (LT). Among patients with end-stage liver disease (ESLD), conventional resting echocardiography may inadequately characterize the hemodynamic burden of provoked LVOT obstruction, resulting in incomplete cardiovascular risk stratification before transplantation. This report details the use of an invasive preload-provocation fluid challenge to distinguish dynamic from fixed LVOT obstruction, thereby directly impacting anesthetic planning and transplant candidacy.

A 69-year-old female patient with drug-induced ESLD displayed a resting transthoracic LVOT gradient of 84 mmHg while on beta-blocker therapy. Cardiac catheterization revealed a baseline intracavitary mid-cavity-to-LVOT gradient of 60 mmHg. Administration of a 500 mL 5% albumin preload over 10 minutes resulted in a reduction of the gradient to 16 mmHg, confirming a load-dependent dynamic obstruction consistent with ESLD physiology rather than fixed LVOT obstruction. Following reassessment, the patient successfully underwent LT utilizing advanced invasive hemodynamic monitoring. Anesthetic management prioritized afterload support and meticulously controlled volume administration, yielding intraoperative cardiovascular stability. Postoperatively, echocardiography demonstrated resolution of LVOT gradients and preserved graft function at six months.

This proof-of-concept case underscores the utility of a standardized invasive preload challenge in distinguishing dynamic from fixed LVOT obstruction and guiding perioperative strategy. In the presence of preload-responsive gradients and careful afterload maintenance, LVOT obstruction should not be considered an absolute contraindication to LT. Integration of this diagnostic approach into pre-transplant assessment protocols may refine cardiovascular risk stratification and expand LT eligibility among patients presenting with this unique hemodynamic profile.

## Introduction

Left ventricular outflow tract (LVOT) obstruction is defined by a peak instantaneous gradient from the left ventricle (LV) to the aorta of at least 30 mmHg [1,2]. Obstruction may be classified as fixed, resulting from structural narrowing of the LVOT, or dynamic, characterized by partial occlusion of the LVOT during systole, frequently associated with systolic anterior motion (SAM) of the mitral valve [3,4]. Elevated LVOT gradients are conventionally assessed using transthoracic echocardiography (TTE) or dobutamine stress echocardiography, employing continuous wave Doppler interrogation across the LVOT.

In patients demonstrating severe LVOT gradients (>50 mmHg), reductions in preload and afterload, tachycardia, and heightened inotropic states may significantly impair LV stroke volume [5]. Consequently, LVOT obstruction presents substantial perioperative risks, as anesthetic and surgical maneuvers can precipitate adverse hemodynamic states.

Within the context of liver transplantation (LT), the perioperative significance of LVOT obstruction has been principally studied in cases of dynamic obstruction, which are associated with increased intraoperative hemodynamic instability; however, evidence does not demonstrate a significant correlation with cardiovascular complications or postoperative mortality [6-8]. By contrast, pronounced LVOT gradients attributable to hypertrophic obstructive cardiomyopathy (HOCM) during LT have been linked to increased morbidity and mortality [9].

This report describes the use of an invasive preload-provocation fluid challenge to evaluate dynamic LVOT obstruction prior to LT. The proof-of-concept case illustrates that a brief, reproducible invasive preload challenge can discriminate dynamic from fixed LVOT obstruction, thereby informing anesthetic technique and transplant eligibility. When LVOT gradients show preload responsiveness and afterload is adequately supported, LVOT obstruction should not be viewed as an absolute contraindication to LT. Integrating this protocol into pre-LT assessment may enhance risk stratification and broaden LT accessibility for patients with this distinct cardiovascular profile.

## Case presentation

A 69-year-old female patient with a six-week history of subacute hepatic failure attributed to nitrofurantoin-induced liver injury was evaluated for LT. Her medical history was notable for ulcerative colitis and atrial arrhythmias, managed with oral mesalazine at a dosage of 3 grams daily and flecainide at 100 mg twice daily, respectively. Upon admission, her Model for End-Stage Liver Disease-Sodium (MELD-Na) score was 33. The MELD-Na score, which ranges from 6 (least severe) to 40 (most severe), is a validated, objective prognostic index employed to quantify liver dysfunction and estimate short-term mortality risk in LT candidates.

**Figure 1:**
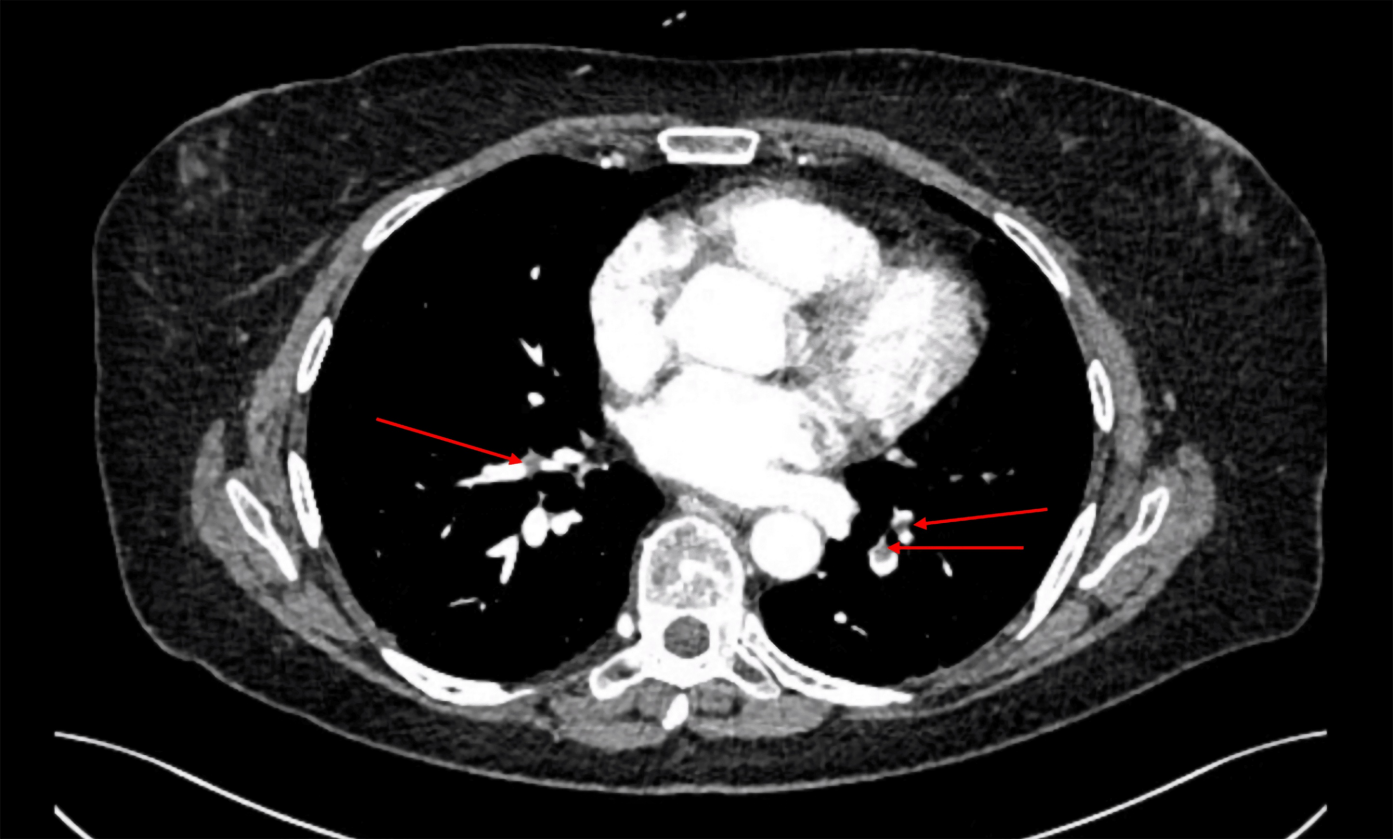
Axial image from CTPA that demonstrates bilateral segmental pulmonary emboli. CTPA: computed tomography pulmonary angiography

The patient presented with decompensated hepatic failure and progressive hepatic encephalopathy, warranting transfer to the intensive care unit (ICU) for rigorous monitoring and supportive therapy. During the ICU course, she experienced type 1 respiratory failure, presumed secondary to aspiration. Axial computed tomography pulmonary angiography (CTPA) identified bilateral segmental pulmonary emboli (Figure 1), prompting initiation of therapeutic anticoagulation. 

Subsequently, the patient developed acute-on-chronic subdural hemorrhages, attributed to anticoagulation therapy, necessitating embolization of the middle meningeal arteries. She also experienced substantial fluid overload, which was managed with diuretic therapy and serial large-volume paracenteses. Laboratory investigations revealed marked thrombocytopenia, coagulopathy, hyperbilirubinemia, and abnormal liver function tests (Table 1). Her blood group was determined to be Type A, RhD positive.

**Table 1:** Laboratory findings immediately prior to liver transplantation

Laboratory test (units)	Laboratory value	Normal range
Hemoglobin (grams/liter)	76	115 –155
White cell count (x10^9/liter)	7.4	4.0 – 12.0
Platelet (x10^9/liter)	44	150 – 400
Prothrombin time (PT) (seconds)	29	11.0 – 15.0
International normalized ratio (INR)	2.4	0.8 – 1.2
Activated partial thromboplastin time (APTT) (seconds)	68	22 – 38
Sodium (mmol/liter)	146	135 – 145
Potassium (mmol/liter)	4	3.5 – 5.2
Chloride (mmol/liter)	110	95 – 110
Bicarbonate (mmol/liter)	27	22 – 32
Urea level (mmol/liter)	14.7	3.5 – 8.0
Creatinine level (micromol/liter)	74	45 – 90
Calcium level (mmol/liter)	2.1	2.10 – 2.60
Magnesium level (mmol/liter)	1.03	0.7 – 1.10
Phosphate level (mmol/liter)	0.7	0.75 – 1.50
Bilirubin total (micromol/liter)	395.2	< 21.0
Alanine aminotransferase (ALT) (units/liter)	48	5 – 35.0
Aspartate Aminotransferase (AST) (units/liter)	64	5 – 30
Gamma-glutamyl transferase (GGT) (units/liter)	47	5 – 35
Alkaline phosphatase (ALP) (units/liter)	164	30 – 110
Albumin level (grams/liter)	28	35 – 52
Ammonia level (micromol/liter)	51	18 – 72

During the course of her critical illness, the patient experienced a syncopal episode characterized by abnormal respiration and absence of a palpable pulse, findings consistent with cardiac arrest. Immediate cardiopulmonary resuscitation (CPR) was administered for one minute, adhering to contemporary resuscitation guidelines to preserve cerebral and myocardial perfusion. The patient demonstrated rapid clinical recovery following the provision of supplemental oxygen and a 500 mL crystalloid bolus; the event was clinically presumed to be vasovagal in origin.

**Figure 2:**
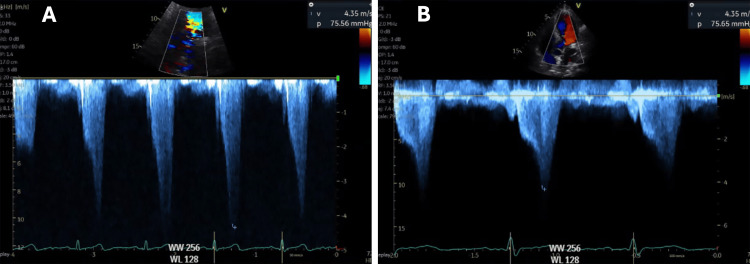
Images A and B show two transthoracic echocardiography (TTE) continuous-wave Doppler echocardiography images from the patient before liver transplantation, obtained across the left ventricular outflow tract (LVOT). Both recordings demonstrate a peak systolic velocity of 4.35 m/s, corresponding to a calculated peak pressure gradient of approximately 75 mmHg, in agreement with significant LVOT obstruction.

Subsequent transfer to the ICU facilitated urgent TTE, which revealed pronounced mid-cavity and dynamic LVOT obstruction. Echocardiographic findings included mid-systolic apposition of hypertrophied septal and lateral walls resulting in an “hourglass” or “dumbbell”-shaped left ventricular cavity, with mid-cavity resting and post-Valsalva continuous-wave Doppler gradients of 75 mmHg and 91 mmHg, respectively (Figure 2).

**Figure 3:**
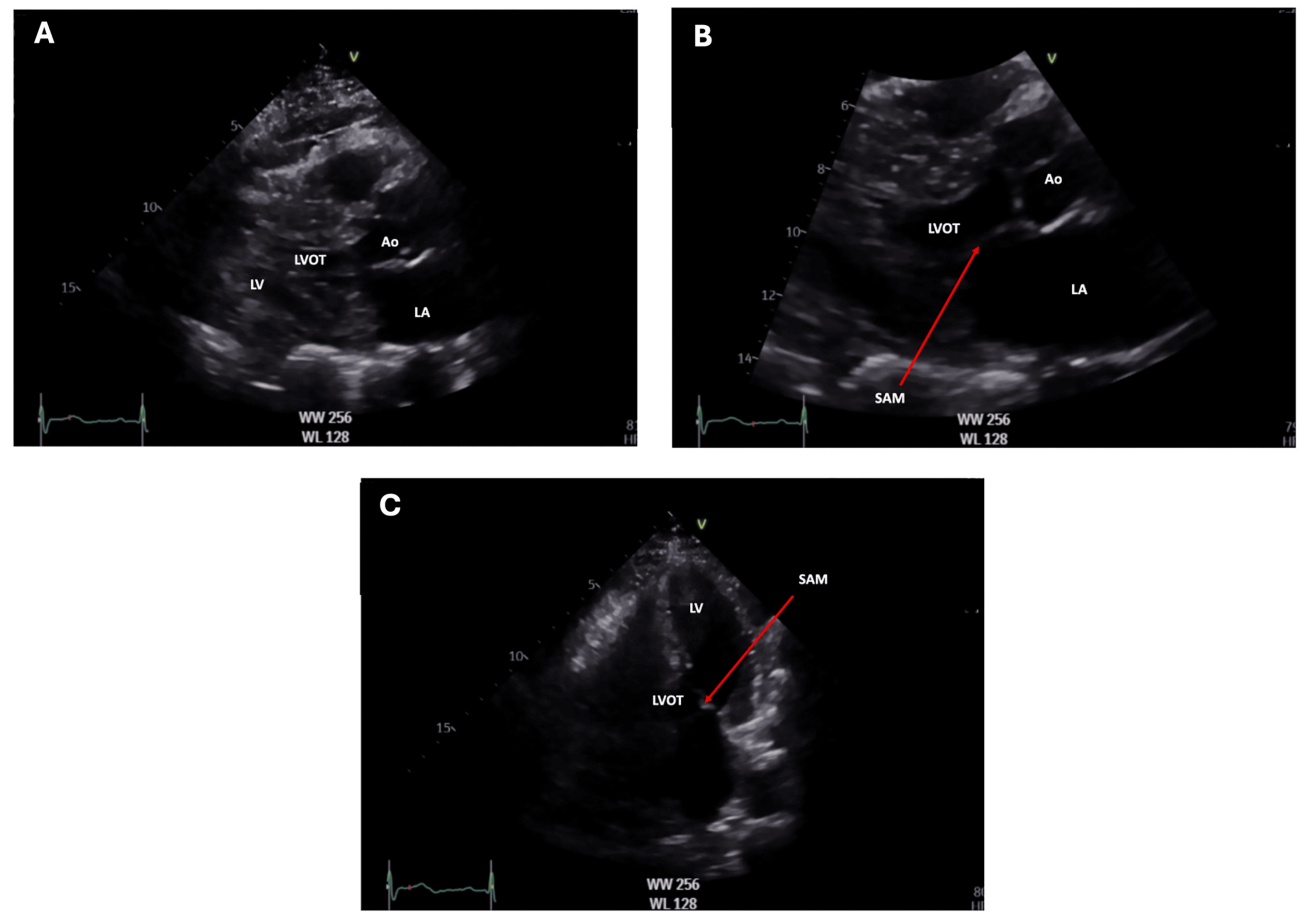
Transthoracic echocardiography (TTE) views of the patient before liver transplantation. Image A demonstrates a parasternal long axis (PLAX) view during late systole. Image B shows the PLAX view with systolic anterior motion (SAM) of the mitral valve during late systole. Image C shows the apical five-chamber view during mid-systole.

Color Doppler demonstrated flow acceleration within the mid-cavity, and the Doppler spectral profile was “dagger-shaped”. SAM of the mitral valve was present (Figure 3), alongside moderate septal (13 mm) and posterior (18 mm) left ventricular wall thickness and a hyperdynamic LV (ejection fraction 76%). Right ventricular size and function were preserved. Additional findings included mild tricuspid regurgitation (TR Vmax 2.2 m/sec), a right ventricular systolic pressure of 20 mmHg plus right atrial pressure, mild mitral and aortic regurgitation, and grade 1 diastolic dysfunction (E/A ratio 0.59). Notably, a transthoracic echocardiogram performed 30 months earlier demonstrated preserved biventricular function and did not meet international guideline-based diagnostic criteria for HOCM (Table 2) [2].

**Table 2:** Diagnostic criteria for hypertrophic obstructive cardiomyopathy (based on international guidelines for the diagnosis of hypertrophic cardiomyopathy). LV: left ventricle; LVOT: left ventricular outflow tract; SAM: systolic anterior motion; IVS: interventricular septum; TDI: tissue Doppler imaging; LA: left atrial; LAVI: left atrial volume index; AF: atrial fibrillation This table presents collated data from reference [2].

Parameter	Criteria/Cutoff	Notes
LV wall thickness	≥15 mm (≥13 mm in first-degree relatives)	In the absence of abnormal loading conditions
Septal/posterior wall ratio	>1.3 (≥1.5 in hypertensive patients)	Indicates asymmetrical hypertrophy
Distribution of hypertrophy	Asymmetric or focal hypertrophy	RV free wall hypertrophy ≥7 mm, reverse IVS curvature
Anterior mitral leaflet length	>30 mm (17 mm/m² indexed)	Evaluates leaflet elongation
Posterior mitral leaflet length	>15 mm	
Papillary muscle abnormalities	Anterior displacement or insertion to anterior leaflet	
SAM of the mitral valve	>30% systolic contact with IVS	Systolic anterior motion
LVOT/Midventricular gradient	>30 mmHg at rest or with provocation	“Dagger” Doppler profile
Systolic function	Lateral S (TDI) <4 cm/s	Impaired longitudinal function
Diastolic function	Lateral e’ <4 cm/s, E/A ratio and filling pressure	LAVI >34 mL/m², E/e’ ratio >10 (less specific)
LA size/volume	LAVI >34 mL/m², LA diameter >45 mm	Predictive of AF and stroke
Apical aneurysm/shape	Hourglass/LV aneurysm	Higher risk of arrhythmia/thromboembolism

Beta-blocker therapy was commenced and titrated to metoprolol 75 mg twice daily over a two-week period, aimed at facilitating echocardiographic improvement. The patient maintained hemodynamic stability with ongoing diuretic management. Nevertheless, follow-up transthoracic echocardiography demonstrated worsening obstruction, evidenced by a peak resting LVOT gradient of 84 mmHg and an augmented gradient of 110 mmHg with the Valsalva maneuver, accompanied by pre-systolic aortic valve closure.

**Figure 4:**
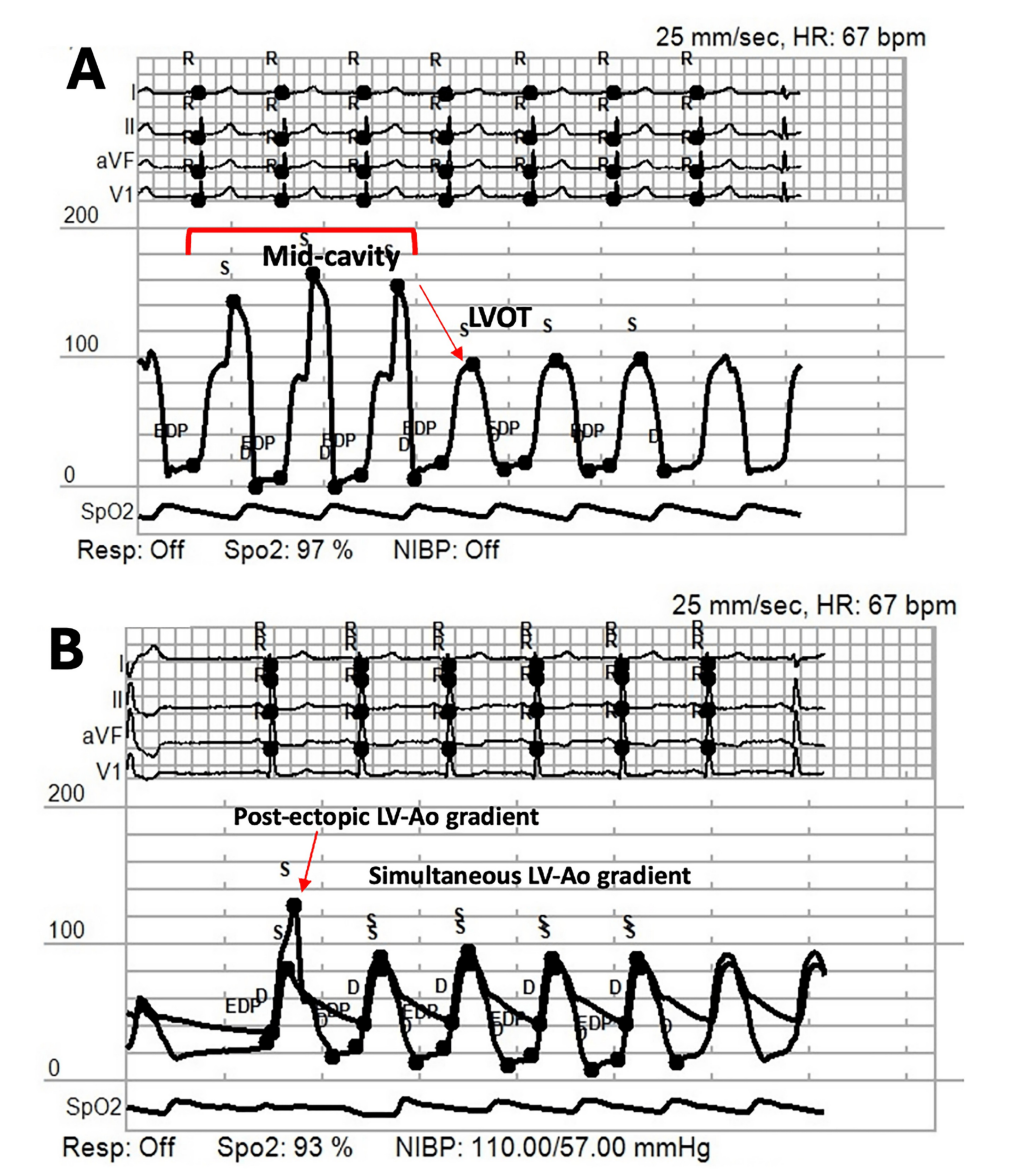
Hemodynamic data from the invasive whole-heart study before liver transplantation. The top panel (A) demonstrates a left ventricular (LV) mid-cavity to left ventricular outflow tract (LVOT) pullback gradient, and the lower panel (B) shows the LV to aorta (Ao) simultaneous gradient after albumin administration. HR: heart rate; Resp: respiratory rate; NIBP: non-invasive blood pressure; SpO_2_: oxygen saturations

To further characterize the LVOT gradient, an invasive whole-heart assessment was performed utilizing a standardized hemodynamic fluid challenge. With the patient positioned supine, a 5 French sheath was placed via the right radial artery, and an end-hole catheter was advanced to the left heart, an approach selected to minimize arterial punctures given the patient’s elevated international normalized ratio (INR). Simultaneous left ventricular and aortic pressure measurements were obtained using a Langston® Dual Lumen Catheter (Vascular Solutions, Inc., Minneapolis, MN) equipped with two transducers. The initial maximal instantaneous gradient from the mid-cavity to the LVOT measured approximately 60 mmHg. Following intravenous infusion of 500 mL of 5% albumin over 10 minutes, a substantial decrease in the maximal instantaneous left ventricular-to-aortic gradient was observed (16 mmHg, post-ectopic), with the average gradient falling to 5 mmHg (Figure 4). These findings suggest that the pronounced LVOT gradient originated from decreased afterload due to liver failure, rather than fixed obstruction. While the mid-cavity outflow gradient increased perioperative risk, it was not classified as an absolute contraindication to LT, and the patient was listed for category 1 transplantation in accordance with the Transplantation Society of Australia and New Zealand (TSANZ) and Australasian Donation & Transplant Coordinators Association (ADTCA) categorization protocols for LT.

Three days post listing, the patient underwent orthotopic LT utilizing a standard piggyback technique with preservation of the inferior vena cava. Intraoperative management incorporated advanced hemodynamic monitoring, including pulmonary artery catheterization (PAC) and TEE, to guide fluid therapy and enable continuous assessment of LVOT gradient, SAM of the mitral valve, and mitral regurgitation.

The perioperative approach was informed by classic pathophysiologic principles used in hypertrophic cardiomyopathy crises, i.e., “full, slow, tight, and less squeeze”, and these strategies were specifically adapted for the transplant context (Table 3) [10]. Sequential TEE evaluations demonstrated progressive widening of the LVOT diameter and resolution of the characteristic dagger-shaped Doppler waveform, substantiating that the adopted regimen of preload augmentation, heart rate moderation, afterload support, and avoidance of excessive inotropy effectively relieved dynamic LVOT obstruction and facilitated uneventful transplantation. The procedure lasted 6.3 hours, with an estimated blood loss of 3.4 liters. Total intraoperative fluid requirements included four units of packed red blood cells (Type O, RhD positive), 2.3 liters of autologous blood, 3.6 liters of balanced crystalloid solution, and 600 mL of 20% albumin.

**Table 3:** Intraoperative management principles are deliberately integrated to relieve dynamic dynamic left ventricular outflow tract (LVOT) obstruction during orthotopic liver transplantation (based on international guidelines for the management of hypertrophic cardiomyopathy). LV: left ventricular; SAM: systolic anterior motion; LVOT: left ventricular outflow tract; TEE: transesophageal echocardiography; CO: cardiac output; CVP: central venous pressure; SVR: systemic vascular resistance; MAP: mean arterial pressure Source: [10]

Hemodynamic principle	Physiological goals	Intra-operative management	Hemodynamic rationale	Monitoring used
“Full”: optimize preload	Keep the LV cavity well filled to diminish SAM and the LVOT gradient	Guided crystalloid/albumin boluses during all phases, according to TEE and continuous CO data	Larger end-diastolic volume widens the LVOT and lessens dynamic obstruction	TEE: LV end-diastolic area; pulse-contour CO; CVP trend
“Slow”: avoid tachycardia	Lengthen diastolic filling time and decrease contractility	Maintained heart rate at 60–70 bpm with balanced volatile anesthesia; esmolol kept ready but not required	Slower rate augments preload and limits SAM-provoking hypercontractility	Five-lead electrocardiogram; arterial-pressure waveform variability
“Tight”: preserve afterload	Sustain SVR to prevent LV emptying	Norepinephrine infusion throughout (up to 10 µg/min pre-reperfusion, 20 µg/min post-reperfusion); ephedrine 6 mg × 2 and metaraminol 0.5 mg × 3 as rescue	Higher SVR raises aortic pressure, opposes the LVOT gradient, and supports coronary perfusion	Invasive arterial pressure; calculated SVR from the CO monitor
“Less squeeze”: avoid excessive inotropy	Minimize a hypercontractile state that narrows LVOT	When vasoplegia persisted, epinephrine 10 µg aliquots (total 100 µg), then infusion ≤5 µg/min, used primarily for α-adrenergic vasoconstriction; the dose titrated down within 30 min after the gradient resolved	Predominant α-effect restores SVR; careful titration prevents β-mediated inotropy that could worsen obstruction	TEE Doppler: disappearance of dagger-shaped LVOT flow; MAP target ≥70 mmHg

**Figure 5:**
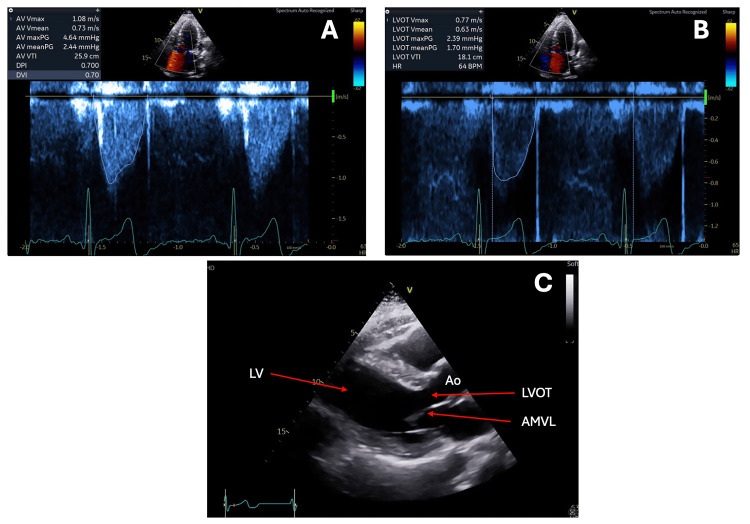
Transthoracic echocardiography (TTE) views obtained six months post-liver transplantation. (A) Parasternal long-axis (PLAX) view in late systole demonstrating absence of systolic anterior motion of the mitral valve. No mid-ventricular cavity obstruction is observed. (B) Pulse-wave Doppler interrogation at the left ventricular outflow tract (LVOT) level revealing normal flow velocities without evidence of obstruction. Continuous-wave Doppler assessment demonstrates normal gradients across the LVOT, mid-cavity, and aortic valve.

The patient’s postoperative course was uncomplicated. Postoperative TTE performed seven days post LT demonstrated resolution of the mid-cavity LVOT gradient, and the cardiac output remained adequate. The patient was discharged home 17 days post transplant. At the six-month follow-up, she reported no cardiovascular or graft-related symptoms. Liver function tests and immunosuppressant levels were within therapeutic ranges (Table 4). Contrast-enhanced abdominal MRI confirmed normal graft vasculature and parenchymal enhancement without evidence of rejection or vascular complications. Repeat TTE revealed complete resolution of the mid-cavity LVOT gradient (peak gradient 6 mmHg, mean 3 mmHg), with normalization of LVOT flow velocity (1.3 m/sec) (Figure 5). Her LV ejection fraction remained preserved at 67%, and her LVH had regressed (interventricular septal thickness 10 mm, compared with 14 mm preoperatively). No structural abnormalities, valvular dysfunction, or diastolic impairment were observed, and her right ventricular systolic pressure was within normal limits.

**Table 4:** Liver function tests and immunosuppressant levels at the time of discharge.

Pathology (units)	Result	Normal Range
Bilirubin total (micromol/liter)	8.6	< 21
Alanine Aminotransferase (ALT) (units/liter)	25	5 – 35.0
Aspartate Aminotransferase (AST) (units/liter)	21	5 – 30
Gamma-glutamyl Transferase (GGT) (units/liter)	35	5 – 35
Alkaline phosphatase (ALP) (units/liter)	97	30 – 110
Albumin level (grams/liter)	37	35 – 52
Tacrolimus level (nanograms/microliter)	9.7	5 – 15

## Discussion

We describe an atypical approach to assess suitability for LT by using an intracardiac study to investigate the responsiveness of a resting and dynamic mid-LV outflow tract gradient to a fluid challenge. This proof-of-concept case illustrates that an invasive preload-provocation fluid challenge can differentiate dynamic from fixed LVOT obstruction, directly informing anesthetic strategy and transplant candidacy. When gradients are preload-responsive and afterload is actively maintained, LVOT obstruction need not be an absolute contraindication to LT. In this case, intracardiac transducers were used to precisely measure the immediate effects of fluid administration on the LVOT gradient, thereby providing sufficient hemodynamic information to guide the treating team’s decision to proceed with surgery.

Our choice of an invasive fluid challenge was driven by the imperative for diagnostic accuracy, patient safety, and rigorous perioperative risk stratification in this high-risk context. While passive leg raising (PLR) is a validated, noninvasive tool for predicting fluid responsiveness, its reliability may be compromised in advanced liver disease due to factors such as severe hypovolemia, increased intra-abdominal pressure, immobility, or altered venous compliance, and echocardiographic limitations. Similarly, although strategies like end-expiratory occlusion testing in ventilated patients offer reversible, noninvasive preload shifts, their performance may be suboptimal in this setting. 

In alignment with perioperative and transplant cardiology guidelines, we undertook invasive, monitored fluid challenges for three primary reasons: i. superior hemodynamic resolution, enabling accurate assessment of filling pressures and detection of dynamic LVOT gradients, which is critical in the cirrhotic population; ii. diagnostic superiority for unmasking preload-sensitive, dynamic LVOT obstruction in HOCM, particularly when noninvasive imaging is inconclusive; and iii. individualized risk stratification to mitigate catastrophic intraoperative complications, supporting optimal outcomes for vulnerable LT candidates. To enhance reproducibility and comparability across studies, we recommend that preload challenge protocols used to assess the effects of fluid administration on the LVOT gradient be rigorously standardized. The implementation of harmonized protocols will promote methodological consistency, enable robust cross-study analyses, and support the development of evidence-based clinical guidance. Our institution’s recommended invasive whole-heart assessment protocol and patient selection criteria to ensure both safety and interpretability are summarized in Table 5.

**Table 5:** Invasive whole-heart standardized preload challenge protocol and patient considerations

Variables	Details
Limitations and contraindications	Volume overload: In patients with renal impairment, advanced heart failure, fluid administration (even as a challenge) may precipitate decompensation.
	Safety in liver failure: While albumin is preferred in cirrhosis to limit sodium, studies show diminished discriminative power of the mini-fluid challenge in advanced Child B or C cirrhosis; increased vascular permeability may also render results less reliable.
	Hemodynamic instability: In patients with marked hypotension, dynamic LVOT obstruction, or at risk for arrhythmias, rapid fluid administration can abruptly alter gradients and is potentially hazardous.
	Infection risk: Invasive arterial monitoring protocols require meticulous aseptic technique and may be contraindicated in the presence of local or systemic infection.
	Technical constraints: Accurate pressure measurements depend on appropriate transducer leveling and calibration; body habitus, vascular access issues, or catheter malposition can affect accuracy.
Patient positioning	Ensure patient is in a supine position and on controlled mechanical ventilation if intubated, with tidal volume set at 6–8 mL/kg ideal body weight
Fluid type and dose	Use of 5% albumin is appropriate for avoiding large sodium loads and minimizing risk in cirrhotic patients; 500 ml is a commonly used volume at out instititution, but mini-fluid challenges (e.g., 150 ml over 1 minute followed by remaining 350 ml over 15 minutes) can also be considered in select high-risk liver disease populations.
Infusion rate	The total volume (e.g., 500 ml) is typically administered over 10–15 minutes to standardize hemodynamic response and prevent rapid volume overload.
Measurement timing	Hemodynamic indices (e.g., pressure gradients) should be measured at baseline, immediately post-challenge, and after completion of the full albumin infusion.
Device and technique	Clearly describe catheter type (e.g., Langston® Dual Lumen Catheter), transducer placement, and acquisition protocols for simultaneous LV and aortic pressure measurements.
Safety and documentation	Monitor the patient for signs of fluid overload, pulmonary edema, arrhythmias, and other complications. Record all protocol steps, findings, and any adverse events for reproducibility

Our patient did not meet the diagnostic criteria for HOCM, had no structural abnormalities, and showed no improvement with beta-blockade therapy. The hyperdynamic left ventricle and the 50% decrease in the LVOT gradient after albumin administration in the setting of severe end-stage liver disease (ESLD_ suggested that decreased afterload was the key factor exacerbating the elevated gradient in our patient. We believed that a new liver graft would restore afterload and improve the gradient over time. The decision-making process to proceed with surgery considered the profound mitigation of LVOT gradient with afterload augmentation from fluid administration. This finding suggested that intraoperative advanced hemodynamic monitoring, including PAC and TEE, could potentially inform appropriate intraoperative management to maintain LV output. This process included observing the LVOT gradient, monitoring for mitral SAM and regurgitation, and optimizing fluid status.

Intraoperative management during LT in patients with LVOT obstruction poses considerable challenges regarding fluid management and vasoactive or inotropic medication administration. ESLD can cause hyperdynamic circulation with elevated cardiac output in response to diminished systemic vascular resistance [5,11]. Each phase of LT (pre-anhepatic, anhepatic, and reperfusion) poses distinct hemodynamic challenges that often require advanced monitoring techniques such as transesophageal echocardiography (TOE) and PAC [12,13]. Classically, the pre-anhepatic phase is prone to substantial fluid shifts and blood loss and may be associated with diminished pre-load, afterload, and compensatory tachycardia [12,14]. The anhepatic phase can further decrease pre-load secondary to clamping of the inferior vena cava and diminished clearance of vasoactive substances [12,13]. The reperfusion phase may initially be associated with decreased afterload, owing to “post-reperfusion syndrome” from the release of cold, acidotic, and inflammatory cytokines [15,16]. As shown in this case, the favorable outcomes observed in patients with dynamic LVOT obstruction undergoing LT [8] might be explained by resolution of hyperdynamic circulation and increased pre-load and afterload after successful LT [5,11,17].

## Conclusions

We present a complex case of a significant resting and dynamic LVOT gradient, which was likely to have been attributable to diminished afterload in the setting of ESLD. An invasive preload-provocation fluid challenge differentiated dynamic from fixed LVOT obstruction, directly informing anesthetic strategy and transplant candidacy. Our findings suggest that dynamic LVOT obstruction in ESLD, when properly characterized and managed with advanced hemodynamic monitoring, should not represent an absolute contraindication to LT. This approach may help optimize risk stratification and expand transplant eligibility for patients with this challenging hemodynamic phenotype.
